# Impact of Power Output on Muscle Activation and 3D Kinematics During an Incremental Test to Exhaustion in Professional Cyclists

**DOI:** 10.3389/fspor.2020.516911

**Published:** 2021-03-10

**Authors:** Camille Pouliquen, Guillaume Nicolas, Benoit Bideau, Nicolas Bideau

**Affiliations:** ^1^M2S Laboratory (Movement, Sports & Health), University Rennes 2, ENS Rennes, Bruz, France; ^2^MIMETIC - Analysis-Synthesis Approach for Virtual Human Simulation, INRIA Rennes - Bretagne Atlantique, Rennes, France

**Keywords:** kinematics, cycling, electromyography, muscle coordination, incremental test

## Abstract

This study aimed to quantify the influence of an increase in power output (PO) on joint kinematics and electromyographic (EMG) activity during an incremental test to exhaustion for a population of professional cyclists. The hip flexion/extension and internal/external rotation as well as knee abduction/adduction ranges of motion were significantly decreased at 100% of the maximal aerobic power (MAP). EMG analysis revealed a significant increase in the root mean square (RMS) for all muscles from 70% of the MAP. Gastrocnemius muscles [lateralis gastrocnemius (*GasL*) and medialis gastrocnemius (*GasM*)] were the less affected by the increase of PO. Cross-correlation method showed a significant increase in the lag angle values for *VM* in the last stage compared to the first stage, meaning that the onset of the activation started earlier during the pedaling cycle. Statistical Parametric Mapping (SPM) demonstrated that from 70% MAP, biceps femoris (*BF*), tibialis anterior (*TA*), gluteus maximus (*GM*), and rectus femoris (*RF*) yielded larger ranges of the crank cycle on which the level of recruitment was significantly increased. This study revealed specific muscular and kinematic coordination for professional cyclists in response to PO increase.

## Introduction

The performance of cyclists is closely linked to their individual physiological profiles as well as biomechanical abilities (Grappe, 2009). In cycling, the monitoring of different physiological variables (heart rate, blood lactate concentration, oxygen uptake, etc.) and/or biomechanical variables [pedaling rate, mechanical power output (PO), torque, etc.] has been performed for decades (Lucia et al., 1997; Bentley et al., 2007; Sayers et al., 2012) to assess adaptations to exercise in cyclists. To this aim, two main types of tests are generally employed: constant-load (continuous/without rest periods or intermittent/with rest periods) tests and incremental (increasing load) tests (e.g., Martinez-Valdes et al., 2016). Whereas constant-load tests offer the possibility to evaluate the evolution of physiological, biomechanical, or neuromuscular parameters related to the appearance of fatigue (Lepers et al., 2000; Ansley et al., 2004; Bini et al., 2008; Theurel et al., 2011; Sayers et al., 2012), incremental tests are extensively used to estimate maximal aerobic power (MAP) and combines fatigue effects and power output changes that meet the requirements of competitive road cycling (Dorel et al., 2008; Macdonald et al., 2008; Ettema and Loras, 2009; Bini and Diefenthaeler, 2010). Moreover, a gradual increase of PO during a short test to its maximal may be a way to understand mechanisms of adaptations during longer sport events or training sessions, despite that some differences between field and laboratory evaluation are still discussed (e.g., Pinot and Grappe, 2014). Incremental tests may also be useful in order to prevent overuse injuries in cycling due to inappropriate alignment of the lower limb segments (Bailey et al., 2003; Gregersen and Hull, 2003) and to an increased solicitation of certain muscle groups (Hug and Dorel, 2009). Whereas, physiological evaluation of high-level cycling is commonly performed, the biomechanical adaptations to an incremental pedaling exercise still remain unclear. In fact, few studies used this type of test (Bini et al., 2016; Pouliquen et al., 2018) to analyze the 3D joint kinematics in view of the increase of PO and/or emergence of fatigue. However, because of the stress it generates on athletes, such an exercise may lead to modifications in lower limb coordination. Regarding performance assessment, maximal exercise protocols—such as incremental test—either measure or predict maximum oxygen consumption (${\overset{∙}{V}O}_{2\max}$) and MAP (Hermansen and Saltin, 1969; Zhang et al., 1991; Bentley et al., 2007). From this approach, specific physiological adaptations could be related to expertise, training level, overload training, or fatigue (e.g., Lucia et al., 1998, 2002). Consequences of fatigue can be evidenced through changes in the activation of the lower limb muscles and modifications of the biomechanics of the pedaling movement. When fatigue appears, changing in muscle activation can lead to kinetic/kinematic changes. Indeed, Dingwell et al. (2008) demonstrated that during a constant load test at 100% of the MAP, changes in electromyogram (EMG) median frequencies would precede changes in movement kinematics. Moreover, Bini and Diefenthaeler (2010) studied modifications of ankle, knee, and hip kinematics and kinetics in a sagittal plane during an incremental test to exhaustion. However, the effect of fatigue and the increase of PO out of sagittal plane during exhaustive (incremental) effort in cycling is less frequently documented in the literature. This approach may limit the insights into the relation between kinematic adaptations and fatigue. This idea is supported by Umberger and Martin (2001) who underlined the idea that 2D evaluation may not be sufficient to take 3D deviations into account, more particularly at the ankle joint complex (inversion/eversion) but also for hip and knee joints. Moreover, a recent study of Pouliquen et al. (2018) demonstrated the importance of taking 3D components (including frontal and transverse planes) into account in order to accurately analyze the lower limb motion during cycling. Gregersen and Hull (2003) suggested that the loads responsible for the relatively high incidence of lower limb overuse injuries in road cycling are the non-driving moments at the knee (varus/valgus and internal/external rotation). This idea was supported by Sayers et al. (2012) who showed significant changes in the tibial rotation despite consistent sagittal movement patterns during the drive phase during a 60-min cycling time trial. To bring into light the connection between muscle coordination and kinematic parameters, conventional 3D analysis may be combined with the analysis of EMG activity in the lower limb muscles. In this aim, the analysis of surface EMG amplitude is commonly used to investigate muscle recruitment adjustments during incremental cycling test (Farina et al., 2004a; Macdonald et al., 2008, Martinez-Valdes et al., 2016). Thus, EMG amplitude has been a relevant parameter to assess changes in muscle function (Macdonald et al., 2008) with various parameters such as fatigue (Hautier et al., 2000; Lepers et al., 2000; Dingwell et al., 2008), pedaling rate (Suzuki et al., 1982; Baum and Li, 2003), body position (Chapman et al., 2008), or intensity level (Lucia et al., 1997; Hug et al., 2006). With regard to the increase of PO on EMG, many studies demonstrated an increase of EMG activity level with respect to PO (Ericson, 1986; Lucia et al., 1997; Hug et al., 2006) due to a large number of motor units recruited. Yet, to the best of our knowledge, only Jorge and Hull (1986) analyzed the impact of PO on muscle activation timing. They found no changes that may be due to low level of PO studied. However, some authors demonstrated that muscle activation timing could be influenced by fatigue (Billaut et al., 2005; Sarre and Lepers, 2005). Regarding cycling tests, contradictory findings emerge from the literature including temporal approaches, spectral analysis techniques, and multi-channel recordings (Farina et al., 2004a; Macdonald et al., 2008; Sbriccoli et al., 2009a; Lenti et al., 2010; Lima da Silva et al., 2018). These discrepancies regarding the effect of incremental exercises on EMG parameters could be partially attributed to the different protocols applied, in particular with regard to the continuous or intermittent nature of the tests (Martinez-Valdes et al., 2016). However, it has been demonstrated that EMG amplitude is sensitive to load changes and the fatigue effects during incremental cycling (Martinez-Valdes et al., 2016). More specifically, it has been suggested that normalized root mean square (RMS) amplitude is the most suitable parameter to monitor changes in muscle activation during continuous incremental cycling exercises, as it was the only variable that was sensitive to both changes in PO and the different fatiguing effects during incremental protocols. Indeed, mean RMS is shown to increase linearly with PO during incremental tests, regardless of the type—intermittent or continuous—of protocol chosen (Macdonald et al., 2008; Travis et al., 2011). In contrast, Martinez-Valdes et al. (2016) demonstrated that instantaneous mean frequency was insensitive to all induced changes in PO and muscle fatigue because of its high variability. Muscle fiber conduction velocity showed great sensitivity to the different fatiguing effects found between protocols, but it cannot be used to evaluate motor unit recruitment all along incremental cycling tests, since it only decreases at a sufficiently high intensity (i.e., close to ${\overset{∙}{V}O}_{2\max}$) (Sbriccoli et al., 2009a; Martinez-Valdes et al., 2016).

As a conclusion, few authors investigated how combined effects of fatigue and PO affect EMG and 3D joint kinematics. Whereas, most studies analyzed kinematics and muscle coordination in a separate way, Dingwell et al. (2008) quantified how changes in muscle fatigue were related to alterations in kinematics, but during a constant load exercise. Recently, Holliday et al. (2019) assessed changes in lower limb EMG magnitudes and 3D kinematics of well-trained cyclists at different exercise intensities. In this context, the aim of this study was to evaluate concomitant changes in muscle activation and 3D kinematics of the lower limb during an incremental cycling test to exhaustion. We set the hypothesis that there would exist a minimal power output from which EMG changes are associated with alterations in 3D root mean square (RMS).

## Methods

### Subjects

Twelve Union Cycliste Internationale (UCI) continental cyclists [(mean ± *SD*) age 25.6 ± 3.8 years; height 1.88 ± 0.06 m; mass 72.5 ± 3.5 kg] volunteered to participate in this experiment after reading and signing an informed consent form in agreement with the local ethics committee and conducted in accordance with the 1975 Declaration of Helsinki. Each cyclist performed the pre-season medical test of the French Cycling Federation. This test is an incremental test to exhaustion pedaling on an SRM indoor trainer (Schoberer Rad Meßtechnik, Jülich, Germany) customized to the participant's own bicycle measurements. A 4-min warm-up at 100 W preceded the incremental step exercise test. This test consisted of a continuous step increase of 50 W every 2 min (starting from 100 W) until exhaustion. All subjects had to maintain a conventional road race position throughout the test, which consisted in the dropped position, hands on drops, arms bent (Faria et al., 2005; Lukes et al., 2005; Chapman et al., 2008). During this incremental exercise, subjects breathed through a face mask and respiratory gas was monitored breath-by-breath (Ultima CardiO2, Medgraphics, St. Paul, MN, USA) that was calibrated prior to each test. The following gas exchange variables were quantified: oxygen uptake (${\overset{∙}{V}O}_{2}$), minute ventilation ($\overset{∙}{V_{E}}$). Heart rate (HR) values were monitored online using a 12-channel ECG (Mortara Instrument Inc., Milwaukee, WI, USA). Expired gas and HR values were averaged every 10 s. The four highest consecutive ${\overset{∙}{V}O}_{2}$ values recorded during the last minute were averaged to determine ${\overset{∙}{V}O}_{2\max}$ (75.2 ± 4.4 ml.min^−1^.kg^−1^ on average for our subjects). The end of the test was based on the achievement of three of the following criteria (Midgley et al., 2007): ${\overset{∙}{V}O}_{2}\;$ plateau (<150 ml.min^−1^ increase), heart rate ≥95% of the age-predicted maximal HR, respiratory exchange ratio (RER) ≥1.1, blood lactate concentration ≥8 mmol.l^−1^, and/or voluntary exhaustion. PO was measured using an SRM training system (Schoberer Rad Meßtechnik, Science version, Germany) with a precision of ±0.5%. Before the experimental procedure, the SRM was calibrated according to the manufacturer's recommendations. The pedaling cadence was monitored all along the incremental test. Cyclists were allowed to self-select their cadence and were instructed to maintain this cadence throughout the test. When cadence dropped by more than 10 rev·min^−1^ for more than 10 s despite strong verbal encouragement, tests were terminated (Karsten et al., 2015). MAP was defined as the PO in the final completed stage of 2 min. All participants fulfilled the exhaustion criteria at the same stage of 350 W corresponding to a test duration of 12 min. Thus, successive stages were 150 W (initial stage), 200, 250, 300, and 350 W (final stage), which correspond respectively to a relative power of 40% (initial stage), 55, 70, 85, and 100% (final stage) of MAP.

### Kinematic Data Acquisition and Processing

Cyclists were equipped with 42 retro-reflective markers placed on anatomical landmarks to calculate the lower limb anatomical rotations following the ISB recommendations (Wu and Cavanagh, 1995; Wu et al., 2002). One marker was placed on each pedal in order to determine during postprocessing bottom dead center (BDC) and top dead center (TDC). Vicon motion capture system (Oxford Metrics, Oxford, UK) was used to record the 3D landmark trajectories. It was composed of 12 high-resolution cameras of four megapixels operating at a nominal frame rate of 100 Hz. The 3D coordinate data of the markers were smoothed using a second-order Butterworth low-pass filter with a cutoff frequency of 10 Hz (Reiser et al., 2002, Sinclair et al., 2014). ROM was calculated for nine anatomical degrees of freedom (DOF): hip, knee flexion/extension (fle./ext.); hip, knee abduction/adduction (abd./add.); hip, knee and ankle internal/external rotation (IR/ER), ankle plantarflexion/dorsiflexion (pla./dor.), and ankle inversion/eversion (inv./eve.). This parameter corresponds of the absolute value of the difference between the minimal and the maximal angle during the cycle. Kinematic data were averaged over 20 crank cycles 1 min after the beginning of each stage.

### Electromyogram Data Acquisition and Processing

The EMG activity of the muscles was recorded using wireless Cometa (Wave Wireless EMG, Cometa, Milan, Italy) at a sampling rate of 1,000 Hz. One rectangular 21 × 41 mm bipolar surface electrode Ag/AgCl with an inter-electrode distance of 20 mm was placed on each of the main right leg muscles: tibialis anterior (*TA*), biceps femoris (long head, *BF*), rectus femoris (*RF*), medialis gastrocnemius (*GasM*), lateralis gastrocnemius (*GasL*), gluteus maximus (*GM*), vastus lateralis (*VL*), and vastus medialis (*VM*). Prior to placing the electrodes, the skin over the muscles was carefully shaved and cleaned using an abrasive cleaner and alcohol swabs to reduce the skin impedance. Each electrode was placed longitudinally with respect to the underlying muscle fiber arrangement, and all of them were located according to the recommendations of surface EMG for non-invasive assessment of muscles (SENIAM; Hermens et al., 2000). EMG signals and kinematic data were synchronized with a Vicon patch panel. Raw EMG signals were pre-amplified with a gain of 1,000 and band-pass filtered at 15–500 Hz through a second-order Butterworth digital filter in order to remove noise or movement interference (DeLuca et al., 2010). EMG analysis was performed using Matlab (MathWorks, Natick, Massachusetts, USA). EMG signals were root mean square (RMS) with a time averaging period of 20 ms. RMS amplitude was normalized by dividing each value by the maximal value of the RMS recording during the test (Rouffet and Hautier, 2008). RMS envelopes were averaged over 20 crank cycles 1 min after the beginning of each stage as for the considered kinematic data. As a key parameter of muscle activation level alterations, the mean RMS of each stage was considered (Table 1). Moreover, cross-correlation was used to measure the angle lag of EMG envelopes for each stage in comparison to the reference stage (150 W), as described in Li and Caldwell (1999). A positive lag angle indicates that the muscle is activated earlier in comparison with the first stage (150 W).

**Table 1:** 3D joint ranges of motion (ROM) presented as means ± *SD* for the five stages of the incremental test.

**Joint**	**DOF**	**Stage (% of MAP)**				
		**40%**	**55%**	**70%**	**85%**	**100%**
Hip	Flexion/Extension (°)	61.8 ± 4.7	61.5 ± 4.9	60.1 ± 5.3	59.8 ± 5.0^**^^(0.39)^	58.8 ± 4.1^**^^(0.44)^
	Abduction/Adduction (°)	11.6 ± 5.1	11.4 ± 5.5	11.4 ± 5.5	11.3 ± 5.8	10.9 ± 5.6
	Internal/External Rotation (°)	21.3 ± 4.8	20.6 ± 4.8	19.8 ± 4.5	19.3 ± 4.8	18.8 ± 5.0^*^^(0.39)^
Knee	Flexion/Extension (°)	87.1 ± 4.3	86.8 ± 4.2	86.2 ± 5.7	86.6 ± 4.5	85.6 ± 3.7
	Abduction/Adduction (°)	13.1 ± 3.4	12.6 ± 3.1	12.4 ± 3.5	12.2 ± 3.2	11.6 ± 2.9^**^^(0.42)^
	Internal/External Rotation (°)	11.8 ± 3.1	12.0 ± 3.3	11.9 ± 3.4	12.1 ± 3.3	11.9 ± 3.6
Ankle	Plantarflexion/Dorsiflexion (°)	17.9 ± 5.9	17.5 ± 6.1	18.0 ± 6.5	18.9 ± 6.4	19.0 ± 5.0
	Inversion/Eversion (°)	13.1 ± 5.1	12.2 ± 4.3	10.9 ± 3.5	11.1 ± 5.2	10.9 ± 4.6
	Internal/External Rotation (°)	11.4 ± 3.7	11.5 ± 3.1	9.0 ± 3.7	8.9 ± 4.8	9.8 ± 4.6

* *When ROM value of the considered stage is significantly lower than 40% of maximal aerobic power (MAP) (p < 0.05)*.

** *When ROM value of the considered stage is significantly lower than 40% of MAP (p < 0.01)*.

*Effect size values of significant difference are shown in brackets*.

### Statistical Analysis

All analyzes were performed using Matlab (MathWorks, Natick, Massachusetts, USA). A Shapiro–Wilk normality test was employed. Kinematic data and EMGrms did not follow a normal distribution, so a Friedman test was used for a one-way repeated measures analysis of variance and a Bonferroni correction for multiple comparisons of the five stages (40, 55, 70, 85, and 100% of MAP), which allowed us to detect significant differences between PO. For the cross-correlation results, multiple comparisons were made using the same methodology. Moreover, Statistical Parametric Mapping (SPM) was performed for EMGrms in order to analyze subgroup differences (Pataky et al., 2013) using the open-source spm1d code (v.M0.4, www.spm1d.org). SPM allowed to objectively identify field regions that co-vary significantly with the increase of PO. Results were expressed as means ± standard deviation (*SD*). The level of significance was set at *p* < 0.05. The common language effect size (ES) was calculated to present the magnitude of the variables that were significantly different after statistical analysis (Hentschke and Stüttgen, 2011). To interpret the results of the ESs, we used the correspondence table with Cohen ES (Cohen, 1988) proposed in the study of Dunlap (1999). The magnitude of the difference was defined as small (ES < 0.61), moderate (0.61 < ES < 0.69), and large (ES > 0.69).

## Results

### Kinematic Results

The effect of PO on ROM is presented in Table 1.

Multiple comparisons demonstrated a significant decrease for the ROM hip fle./ext. at 85% MAP (59.8° ± 5.0°, *p* < 0.01, ES = 0.39) and 100% MAP (58.8° ± 4.1°, *p* < 0.01, ES = 0.44) in comparison to 40% MAP (61.8° ± 4.7°). Moreover, a significant decrease (*p* < 0.05, ES = 0.39) was exhibited for ROM hip int./ext. rotation between 40% MAP (21.3° ± 4.8°) and 100% MAP (18.8° ± 5.0°) as well as ROM knee abd./add. (*p* < 0.01, ES = 0.42) between 40% MAP (13.1° ± 3.4°) and 100% MAP (11.6° ± 2.9°).

### Electromyogram Results

Peak EMGrms for each stage and multiple comparisons between different stages are summarized in Table 2. Table 2 shows that all muscles displayed a significant increase (*p* < 0.01) for each muscle from the third stage (70% MAP) in comparison to the first one (40% MAP). Moreover, this multiple comparison between stages revealed that two successive stages did not provide significant differences. Peak EMGrms demonstrated that *GasL* was the least affected muscle because it demonstrated only four significant differences (70 vs. 40% MAP, 85 vs. 40% MAP, 100 vs. 40% MAP, and 100 vs. 55% MAP, *p* < 0.01).

**Table 2:** Mean ranges of motion (RMS) value of each stage and multiple comparisons.

		**Stages (% of MAP)**				
		**40%**	**55%**	**70%**	**85%**	**100%**
Muscles	BF	0.16 ± 0.05	0.20 ± 0.05	0.25 ± 0.06^a^^(0.15)^	0.30 ± 0.06^a^^(0.09),^^b^^(0.18)^	0.37 ± 0.05^a^^(0.01),^^b^^(0.04),^^c^^(0.10)^
	GM	0.12 ± 0.06	0.16 ± 0.07	0.18 ± 0.08^a^^(0.12)^	0.22 ± 0.07^a^^(0.03),^^b^^(0.11)^	0.28 ± 0.07^a^^(0.01),^^b^^(0.01),^^c^^(0.03)^
	GasL	0.21 ± 0.06	0.25 ± 0.07	0.28 ± 0.08^a^^(0.27)^	0.30 ± 0.07^a^^(0.17)^	0.35 ± 0.05^a^^(0.08),^^b^^(0.18)^
	GasM	0.20 ± 0.06	0.23 ± 0.05	0.26 ± 0.06^a^^(0.36)^	0.29 ± 0.07^a^^(0.28),^^b^^(0.34)^	0.32 ± 0.06^a^^(0.23),^^b^^(0.27)^
	RF	0.11 ± 0.05	0.16 ± 0.07	0.20 ± 0.07^a^^(0.08)^	0.24 ± 0.07^a^^(0.01),^^b^^(0.09)^	0.33 ± 0.06^a^^(0.01),^^b^^(0.01),^^c^^(0.04)^
	TA	0.14 ± 0.04	0.17 ± 0.05	0.22 ± 0.06^a^^(0.20)^	0.28 ± 0.06^a^^(0.04),^^b^^(0.10)^	0.34 ± 0.05^a^^(0.02),^^b^^(0.05),^^c^^(0.12)^
	VL	0.17 ± 0.04	0.21 ± 0.04	0.25 ± 0.04^a^^(0.13)^	0.28 ± 0.05^a^^(0.05),^^b^^(0.16)^	0.33 ± 0.05^a^^(0.01),^^b^^(0.06),^^c^^(0.13)^
	VM	0.20 ± 0.05	0.24 ± 0.06	0.27 ± 0.04^a^^(0.24)^	0.28 ± 0.05^a^^(0.20)^	0.33 ± 0.05^a^^(0.13),^^b^^(0.40)^

a *Significant difference (p < 0.01) for the comparison of a given stage with the 40% of maximal aerobic power (MAP) stage*.

b *Significant difference (p < 0.01) for the comparison of a given stage with the 55% of MAP stage*.

c *Significant difference (p < 0.01) for the comparison of a given stage with the 70% of MAP stage*.

*Effect size values of significant difference are shown in brackets*.

*BF, biceps femoris; GM, gluteus maximus; GasL, gastrocnemius lateralis; GasM, gastrocnemius medialis; RF, rectus femoris; TA, tibialis anterior; VL, vastus lateralis; VM, vastus medialis*.

**Figure 1:**
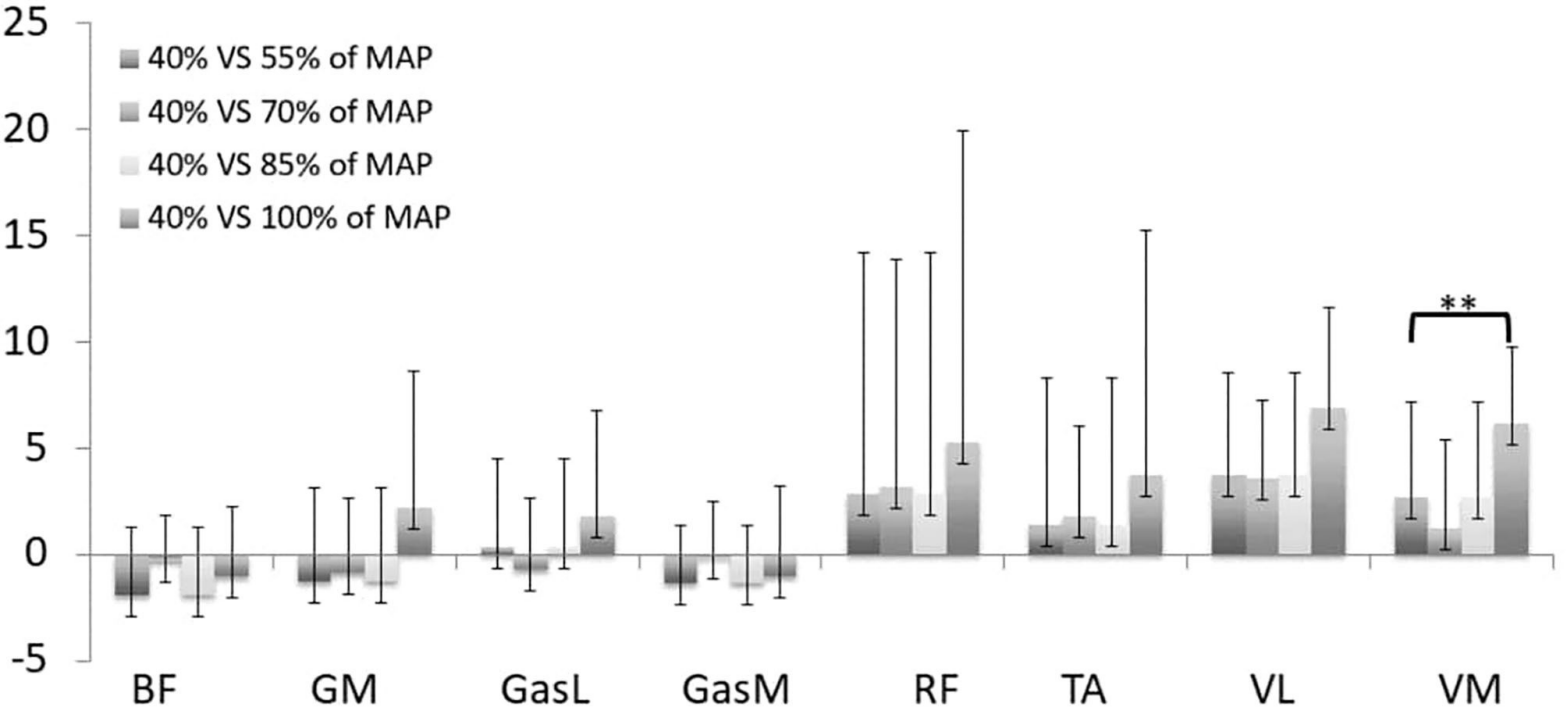
Lag angle k for stages 55% (1), 70% (2), 85% (3), 100% (4) compared to stage 40% of maximal aerobic power (MAP). **p < 0.01 for comparison with the stage 1. TA, tibialis anterior; VM, vastus medialis; VL, vastus lateralis; RF, rectus femoris; GasM, gastrocnemius medialis; GasL, gastrocnemius lateralis; GM, gluteus maximus; BF, biceps femoris. A positive lag angle indicates that the muscle is activated earlier in comparison with the first stage (150 W).

The only difference in multiple comparisons of cross-correlation was obtained in comparison to the first stage (40% MAP). Figure 1 displays the lag angle for each stage compared to the first stage. A positive lag angle indicates that the muscle activation started earlier in comparison to the reference stage. All muscles exhibited a trend toward the increase of the lag angle between the 40% MAP vs. 55% MAP and 100 vs. 40% MAP comparisons. A significant increase (*p* < 0.01, ES = 0.27) was found for the *VM* in the last stage compared to the first stage, demonstrating an earlier pick of the EMG burst.

**Figure 2:**
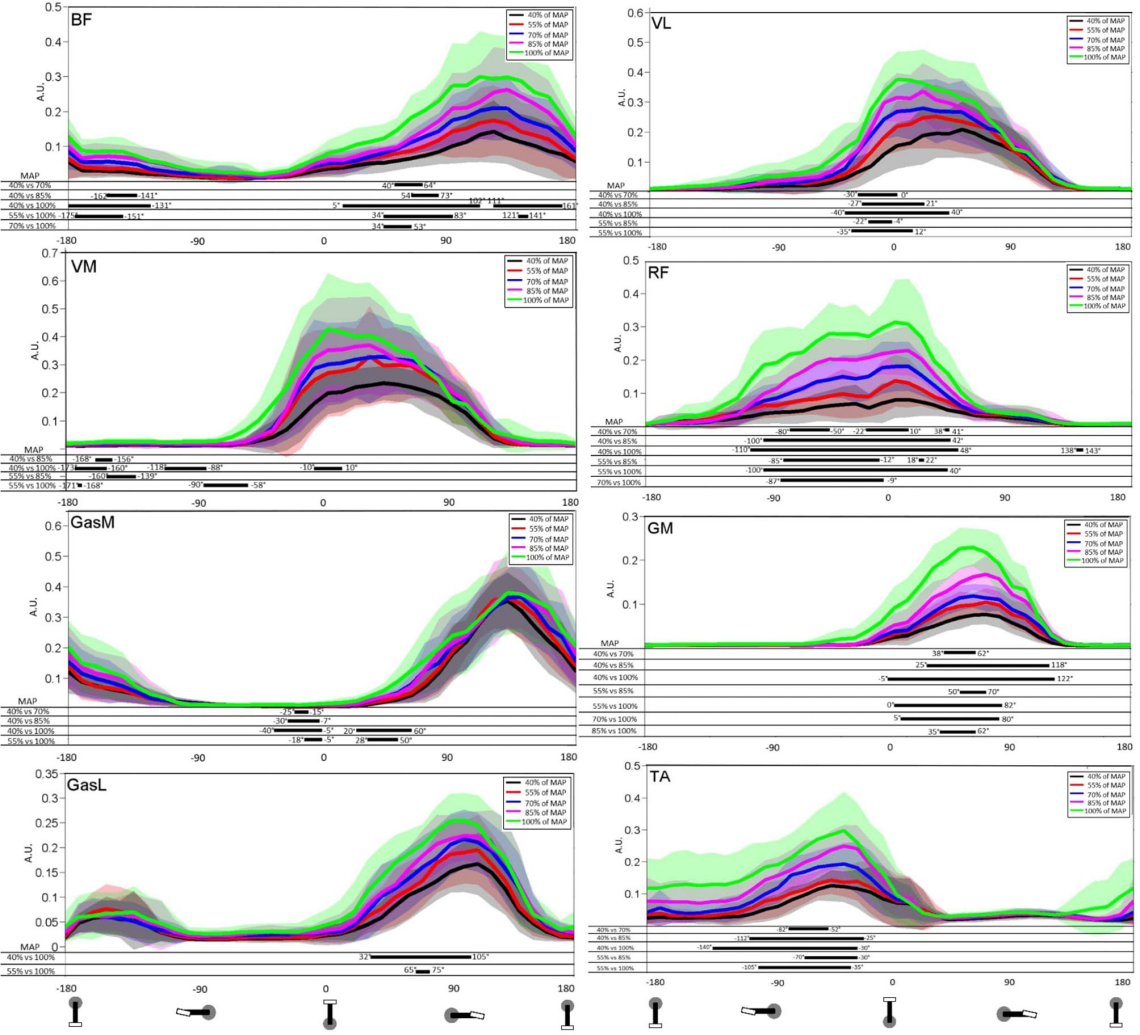
Mean and standard deviation of the root mean square (RMS) of the EMG signal for stages 40, 55, 70, 85, and 100% of maximal aerobic power (MAP). Results are presented from bottom dead center to bottom dead center of the next cycle. TA, tibialis anterior; VM, vastus medialis; VL, vastus lateralis; RF, rectus femoris; GasM, gastrocnemius medialis; GasL, gastrocnemius lateralis; GM, gluteus maximus; BF, biceps femoris. Statistical Parametric Mapping of the differences between stages is presented on the part below each graph.

SPM analysis of the EMGrms (Figure 2) examined differences between each stage for all muscles. This comparison allowed determining spatiotemporal differences in EMGrms envelopes between stages and the impact of PO increase. All muscles demonstrated differences (i.e., higher recruitment) with the increase of PO but not for two consecutive stages. *BF* exhibited significant differences near bottom dead center and during the power phase (70 vs. 40% MAP, 85 vs. 40% MAP, 100 vs. 40% MAP, 100 vs. 55% MAP, and 100 vs. 70% MAP). *VL* exhibited significant differences near TDC (70 vs. 40% MAP, 85 vs. 40% MAP, 100 vs. 40% MAP, 85 vs. 55% MAP, and 100 vs. 55% MAP). *GasL* displayed significant differences during the power phase (70 vs. 40% MAP and 100 vs. 55% MAP). *GasM* showed significant differences near TDC and at the beginning of the power phase (70 vs. 40% MAP, 85 vs. 40% MAP, 100 vs. 40% MAP, and 100 vs. 55% MAP). *GM* demonstrated significant differences during the power phase (70 vs. 40% MAP, 85 vs. 40% MAP, 100 vs. 40% MAP, 85 vs. 55% MAP, 100 vs. 55% MAP, 100 vs. 70% MAP, and 100 vs. 85% MAP). *RF* exhibited significant differences mainly at the end of the recovery phase and at the beginning of the power phase (70 vs. 40% MAP, 85 vs. 40% MAP, 100 vs. 40% MAP, 85 vs. 55% MAP, 100 vs. 55% MAP, and 100 vs. 70% MAP). *VM* presented significant differences all along the recovery phase (85 vs. 40% MAP, 100 vs. 40% MAP, 85 vs. 55% MAP, and 100 vs. 55% MAP). *TA* demonstrated significant differences at the end of the recovery phase (70 vs. 40% MAP, 85 vs. 40% MAP, 100 vs. 40% MAP, 85 vs. 55% MAP, and 100 vs. 55% MAP).

## Discussion

This study examined concomitant changes in kinematics and muscle recruitment induced by an incremental test to exhaustion. Since cyclists were instructed to maintain the pedaling cadence throughout the test and since the posture was fixed (dropped position, hands on drops), such changes were only due to the incremental exercise.

From a kinematic viewpoint, the results of the 3D ROM were similar to previous findings from the literature (Sayers et al., 2012; Bini et al., 2014). The incremental test was associated with the reduction of ROM for three DOF at 100% MAP in comparison with 40% MAP (hip fle./ext., hip int./ext. rotation, and knee abd./add.) and for one DOF (hip fle./ext.) at 85% MAP in comparison with 40% MAP. These results highlight the importance of non-sagittal movements associated with power increase. Simultaneous modifications of the kinematics in different anatomical planes may be due to the movement in closed kinematic chain (Pouliquen et al., 2018) and/or associated with a specific neuromuscular strategy to combat fatigue (Gates and Dingwell, 2008). According to the results of our study, the planar assumption sometimes used in the literature (e.g., Fregly and Zajac, 1995; Bini et al., 2013) may be questionable in the light of significant modifications for some anatomical rotations out of the sagittal plane. Those joint rotations may be harmful to the physical integrity of cyclists (Ruby et al., 1992; Bailey et al., 2003; Abt et al., 2007). In particular, some authors suggested that DOF out of the sagittal plane may be a cause of overuse injuries (Bailey et al., 2003; Gregersen and Hull, 2003; Pouliquen et al., 2018). To better understand the occurrence of kinematic alterations during incremental test, concomitant changes in muscles' recruitment are of great interest.

EMG results demonstrated significant modification with the increase of PO for the recruitment level. A significant increase (*p* < 0.01) of the mean RMS was observed for all stages (from 70% of MAP) compared to the reference stage (40% MAP). This increase is due to supplementary recruitment of motor units force (Edwards and Lippold, 1956) to compensate the decrease in force contraction that occurs in the fatigued muscle fibers in order to balance the difficulty of the considered stages. Modification on the EMGrms induced by an increase of PO is not instantaneous because of the level of expertise of our cyclists; indeed, important repetition of a movement generates a neuromuscular adaptation to the effort on expert population (Takaishi et al., 1998; Hug et al., 2004b; Chapman et al., 2008; Martinez-Valdes et al., 2016). Moreover, peak EMGrms increased in response to muscle fatigue because of multiple parameters: amplification in motor unit discharge rate (Gandevia, 2001), additional recruitment of non-fatigued motor units, and prolongation of intracellular action potentials (Martinez-Valdes et al., 2016). RMS analysis is one of the most interesting variables to understand the impact of fatigue because it is the most sensitive parameter during fatigue cycling exercise (Macdonald et al., 2008).

Analysis of EMG signals by the cross-correlation method exhibited a trend toward the increase of the lag angle during the incremental test. The most significant effect was obtained for *VM*, which illustrates an earlier muscle activation in the pedaling cycle. Such an alteration of activation timing has already been highlighted through an increase of the lag angle for muscle activations and was attributed to the need to produce a higher propulsive force in order to generate the PO (Dorel et al., 2009). However, in our study, angle lag varied positively and negatively during the test, demonstrating that the muscle coordination strategy evolves nonlinearly with the gradual increase of PO. The heterogeneity in angle lag values as a response to PO increase may be related to the level of expertise of the population (Hug et al., 2004a; Dorel et al., 2008). In contrast, gastrocnemius muscles (*GasL* and *GasM*) seem to be the least impacted. These muscles are bi-articular and actuate knee and ankle rotations, which may explain the small changes during the test. Indeed, van Bolhuis et al. (1998) demonstrated that the bi-articular muscles are activated in order to control the direction of the external force and they generate less power during the pedaling movement (Ericson, 1986). This result coincides with previous works related to fatigue test in cycling (Bini et al., 2008; Dorel et al., 2009), which observed that bi-articular muscles are less impacted. Moreover, muscle contraction of *GasM* helped to stabilize the ankle and to transfer force to the pedal (Holliday et al., 2019). Thus, the few modifications in EMG timing may explain the small increase in the ankle pla./dor. ROM (+1.1° between stage 40 and 100% MAP). Whereas, cross-correlations can be used to give an objective estimation of similarities and temporal characteristics of muscle activity patterns, it does not enable to quantify the effect of PO increase on intra-cycle differences in EMGrms envelopes. Therefore, SPM analysis is a powerful method to address this limitation and allows better understanding of the impact of PO increase on muscle coordination.

SPM analysis showed significant differences between the majority of the muscles (*VL, BF, VM, GM, GasM, RF*, and *TA*), with five of them showing a significant difference during the power phase (*VL, GasL, GasM, GM*, and *BF*). Interestingly, SPM analysis revealed that *GM, RF*, and *TA* were modified all over the activation phase, whereas the progression of the test affected the *VL* activation mainly in the beginning of its activation phase. *RF* showed a larger range of the crank cycle on which the level of recruitment was significantly increased for the comparisons 85 vs. 40% MAP and 100 vs. 40% MAP. More specifically, the PO increase generated an increase in *RF* recruitment mainly before (-90°-0°) and slightly after (0°-50°) TDC. While previous studies highlighted an early muscle activation with the increase of PO or velocity (Neptune et al., 1997; Baum and Li, 2003; Turpin et al., 2011a,b; Holliday et al., 2019), the present study offers complementary insights by showing an additional recruitment of *RF* all along its activation phase. Such a result was previously obtained even for a lower level of expertise (Holliday et al., 2019). With regard to the functional role of hip flexion before TDC, driving the knee around TDC and knee extension in the push phase, it has been suggested that such an increase in activation may compensate the inability of other muscles to exert an effective force to the crank rotation (Holliday et al., 2019). Our results showed that the *TA* activation follows the same trend, as illustrated by an increase in response to PO increment in a specific range before TDC (-90° to−40°). As *TA* activation is directly linked to the ability to effectively orient the force, it may contribute to produce a high effective force around TDC (Hug et al., 2008). Moreover, such an increase during this phase may be attributed to modifications in ankle dorsiflexion (Holliday et al., 2019). Regarding *GM*, whose one of its primary function is hip extension, significant differences in the temporal activation range increase during the test going from [38°;62°] (70 vs. 40% MAP) to [-5°;122°] (100 vs. 40% MAP). This increase is due to the need for the cyclist to produce more pedal force in order to maintain the PO. Similar results were found in the study of Blake et al. (2012), where *GM* demonstrated the largest increase during the effort between 25% of ${\overset{∙}{V}O}_{2\max}$ (103.5 W ± 2.3 W) and 90% of ${\overset{∙}{V}O}_{2\max}$ (325.7 W ± 8.7 W). This can be attributed to the fact that *GM* is one of the most important contributors to power production. However, the fact that *GM* showed a significant increase in activation during the last stage should also be considered along with its other primary function as a hip external rotator. Indeed, *GM* is a large muscle that can act as a potent external rotator, especially for low to moderate hip flexion angles. This muscle maintains an external rotation moment arm throughout 0°-90° of flexion, which includes the joint angular sector swept during the pedaling cycle (Delp et al., 1999). These functional features in both sagittal and transverse planes seem to be corroborated by the significant decrease of fle./ext. and IR/ER ROM that were accompanied by a significant increase of activation for this muscle during the last stage. The *VL* muscle, whose primary function is knee extensor, demonstrated a significant increase near TDC with the SPM analysis from [-30°;0°] (70 vs. 40% MAP) to [-40°;40°] (100 vs. 40% MAP). This early recruitment is generally explained by the need to maintain the PO (Holliday et al., 2019). Another strength of the SPM method used in this paper lies in its ability to evaluate more precisely where differences in EMGrms occur all along the pedaling cycle. Six of the eight muscles analyzed (*BF, GasL, TA, RF, GM*, and *VL*) presented an increase in the EMGrms and a significant difference in the SPM analysis during the main burst (Figure 2). This increase of the main burst during the cyclic task is due to fatigue and the increase of PO (Turpin et al., 2011a,b). The increase of PO mainly affected the muscle coordination during the power phase for five muscles (*RF, BF, GM, GasL*, and *GasM*), which are generally regarded as essential in the pedaling cycle. Indeed, four of these muscles (*GasL, GasM, RF*, and *BF*) are bi-articular and are known to be activated in order to transfer energy between joints and to control the direction of force production on the pedal (Van Ingen Schenau et al., 1992).

The modifications in muscle activations also have to be discussed based on their secondary actions. Indeed, while the dominant motions of the lower limb during the pedaling cycle occur in the sagittal plane, there also are significant rotations in the two remaining planes (frontal and transverse) (e.g., Umberger and Martin, 2001; Bini et al., 2016; Pouliquen et al., 2018). Such secondary functions play a crucial part especially when specific angulations occur in the sagittal plane. Concerning the knee, because of its anatomical orientation, the major muscles crossing this joint are traditionally classified as knee joint flexors (hamstring and gastrocnemius) and extensors (quadriceps), but functional loads are not limited to a single axis. Even if it remains difficult to assign angular displacements out of the sagittal plane to a given muscle, the modification of the recruitment of certain muscles having secondary actions outside the sagittal plane can be viewed in light of the concomitant modifications of 3D ROM. Among the eight muscles, *BF*, whose activation was the most significantly increased, may have played a crucial role in non-sagittal movements due to its secondary actions. From an anatomical viewpoint, Dostal et al. (1986) showed that *BF* displays a major moment arm for hip extension but also non-negligible moment arms for hip external rotation and adduction. More recently, using surface EMG during various combinations of sagittal, frontal, and transverse internal joint loadings, Flaxman et al. (2017) showed that *BF* (long head) was associated with hip and knee adduction moments. They hypothesized that such a contribution may arise from an antagonistic force to oppose hip and knee adduction moments and/or an activation profile reflecting a bimodal pattern that encompasses external hip/knee rotation and hip extension/knee flexion. Inferring with the individual muscle contribution to displacements out of the sagittal plane is a challenging task, and this would require solving the redundancy of the musculoskeletal system. In this aim, recent developments of musculoskeletal models showed the interest of including 3D joint configurations in relation to muscle actuations (Erdemir et al., 2007). Even if reliable muscle force output can be obtained for knee-spanning muscles when using a 1 DOF knee joint, a combination of muscle forces must be considered to match knee kinematics in all three planes when more DOF are included in the knee (Mokhtarzadeh et al., 2014). Another solution would be to address the recruitment of muscles that generate movements outside the sagittal plane, but this would have required the collection of invasive EMG data for deep or hard-to-reach muscles.

Owing to the abovementioned elements, technical skills and the ability to maintain the direction of the force production during the power phase seem to be key factors to optimize performance and to prevent injuries (Bini and Hume, 2014). Our study revealed that modifications of kinematic patterns during the incremental test to exhaustion occurred later than peak EMGrms. This time lag of modification may be due to the technical skills and expertise of the population. In fact, kinematic patterns are less prone to alterations, especially at low intensities, unlike muscle coordination. The first significant decrease appeared for the hip fle./ext. at 85% MAP (*p* < 0.01, ES = 0.39). Then, a significant decrease arose also for knee rotation in the frontal plane at 100% MAP (*p* < 0.01, ES = 0.42). This time shift between kinematic and muscular changes has already been reported in the literature during a constant load test (Dingwell et al., 2008) and during a submaximal incremental exercise (Holliday et al., 2019). A classically suggested hypothesis lies in the change of coordinative strategy in order to maintain the effort to achieve (Dingwell et al., 2008). This change in joint coordination related to muscle modification may result from the co-contraction of antagonistic muscles. Indeed, Hirokawa (1991) demonstrated that this phenomenon of co-activation reduces bone displacement and rotations during the movement. In addition, it reduces the pressure distribution on the surface of the joint (Solomonow et al., 1988) and limits the premature occurrence of musculoskeletal injuries. This phenomenon of co-activation varies with the level of expertise. Indeed, Chapman et al. (2008) demonstrated that novice cyclists had higher individual variance and a larger population variance compared to a trained cyclist population for muscle activation measured with Onset/Offset.

This study presents some limitations. Indeed, we analyzed only the muscle coordination and the kinematics of the right leg. It would be interesting afterward to study and compare the bilateral behavior of cyclists in order to provide more complete information (Pouliquen et al., 2018). Another limitation of our study deals with the characteristics of the incremental test used, in particular, the power increments (50 W) and step durations (2'). Thus, despite the relatively homogeneous level of expertise (75.2 ± 4.4 ml.min^−1^.kg^−1^), MAP values could be refined by smaller power increments and step durations, making it possible to better account for interindividual differences in MAP. It should also be kept in mind that the evaluation was conducted 5 weeks after resumption of the training program that could explain the low values of MAP reported in our study, although participants are professional cyclists. It may reasonably be hypothesized that improvements in cycling economy and neuromuscular efficiency may significantly increase MAP values obtained in the current study (Billat et al., 2003). Moreover, a refinement of the stage durations and power increments would allow a better identification of the nonlinear EMG vs. power curve throughout the whole duration of the incremental test. In particular, such developments may help to determine kinematic alterations in relation to EMG thresholds and aerobic and anaerobic thresholds (Lucia et al., 1999; Hug et al., 2003; Briscoe et al., 2014).

An attractive prospect to our study lies in the strict characterization of separate effects of fatigue and PO during the test, which was not the main objective of this paper. Indeed, as the protocol used in our study combines the effects of increased power, fatigue, and duration, it is difficult to attribute changes in joint kinematics and muscle recruitment solely to any of the above factors. Concerning the fatigue effect, interesting perspectives to this work would relate to the use of mean muscle fiber conduction velocity (MFCV) that can be estimated non-invasively from the surface EMG recordings of multiple closely spaced electrodes. Thus, MFCV has been previously used as a tool to infer motor unit recruitment during incremental cycling exercise (Sbriccoli et al., 2009a; Lenti et al., 2010). However, partly contradictory results were reported during fatiguing dynamic contractions such as exhaustive cycling exercise. Indeed, since some studies have reported a decrease in MFCV (Farina et al., 2004b; Sbriccoli et al., 2009b) or unchanged MFCV (Macdonald et al., 2008; Schmitz et al., 2012) in cycling, further investigations would be required.

Finally, this study demonstrates the importance for coaches to consider the 3D features of movement in cycling because some important modifications appear out of the sagittal plane. ROM analysis is a simple indicator that can be easily used by cyclists and trainers during incremental exercise until exhaustion. Moreover, the SPM analysis enables to describe how the progression of the test engenders inter-muscle differences in activation within the crank cycle. Such an approach may indicate which muscles are the most impacted by the incremental test, which may thus provide relevant areas for improvement regarding physical conditioning and pedaling technique.

## Conclusion

This study demonstrated the impact of an incremental test on joint and muscle coordination in professional cyclists. The RMS analysis showed neuromuscular adaptations from the third stage (70% MAP), and the ROM analysis exhibited adaptations only from the fourth stage (85% MAP). SPM analysis demonstrated that the majority of the muscles presented an early activation when PO increased. This result was confirmed by the cross-correlation analysis. These indicators suggested neuromuscular adaptations preceding joint kinematic modifications as the workload increased. The hip flexion/extension and internal/external rotation ROM were the most affected DOF at the final stage of the incremental test. Gasctrocnemius muscles and vastii were slightly sensitive to power increment. Conversely, from 70% MAP, *BF, TA, GM*, and *RF* yielded larger ranges of the crank cycle on which the level of recruitment was significantly increased.

## Data Availability Statement

The raw data supporting the conclusions of this article will be made available by the authors, without undue reservation, to any qualified researcher.

## Ethics Statement

The study involving human participants was reviewed and approved by French Cycling Federation and the local ethics committee. It was conducted in accordance with the 1975 Declaration of Helsinki. The participants provided their written informed consent to participate in this study.

## Author Contributions

CP, NB, BB, and GN were involved in the conceptualization and design of the study, recruited the participants, and collected the data. CP processed and analyzed the data and produced the figures and tables. CP, NB, and GN drafted the manuscript. All authors critically revised the manuscript and approved the final submitted version.

## Conflict of Interest

The authors declare that the research was conducted in the absence of any commercial or financial relationships that could be construed as a potential conflict of interest.

## References

[B1] Abt J. Smoliga J. Brick M. Jolly J. Lephart S. Fu F. (2007). Relationship between cycling mechanics and core stability. J. Strength Condition. Res. 21, 1300–1304. 10.1519/00124278-200711000-00056 18076271

[B2] Ansley L. Schabort E. Gibson A. S. C. Lambert M. Noakes T. (2004). Regulation of pacing strategies during successive 4-km time trials. Med. Sci. Sports Exer. 36, 1819–1825. 10.1249/01.MSS.0000142409.70181.9D 15595306

[B3] Bailey M. Maillardet F. Messenger N. (2003). Kinematics of cycling in relation to anterior knee pain and patellar tendinits. J. Sports Sci. 21, 649–657. 10.1080/0264041031000102015 12875315

[B4] Baum B. S. Li L. (2003). Lower extremity muscle activities during cycling are influenced by load and frequency. J. Electromyogr. Kinesiol. 13, 181–190 10.1016/S1050-6411(02)00110-4 12586523

[B5] Bentley D. Newell J. Bishop D. (2007). Incremental exercise test design and analysis, implications for performance diagnostics in endurance athletes. Sports Med. 37, 575–586. 10.2165/00007256-200737070-00002 17595153

[B6] Billat V. Lepretre P. M. Heugas A. M. Laurence M. H. Salim D. Koralsztein J. P. (2003). Training and bioenergetic characteristics in elite male and female Kenyan runners. Med. Sci. Sports Exer. 35, 297–304. 10.1249/01.MSS.0000053556.59992.A9 12569219

[B7] Billaut F. Basset F. A. Falgairette G. (2005). Muscle coordination changes during intermittent cycling sprints. Neurosci. Lett. 380, 265–269 10.1016/j.neulet.2005.01.048 15862899

[B8] Bini R. R. Dagnese F. Rocha E. Silveira M. Carpes F. Mota C. (2016). Three-dimensional kinematics of competitive and recreational cyclists across different workloads during cycling. Eur. J. Sport Sci. 16, 553–559. 10.1080/17461391.2015.1135984 26783692

[B9] Bini R. R. Diefenthaeler F. (2010). Kinetics and kinematics analysis of incremental cycling to exhaustion. Sports Biomech. 9, 223–235. 10.1080/14763141.2010.540672 21309297

[B10] Bini R. R. Diefenthaeler F. Mota C. Guimaraes A. (2008). Physiological and electromyographic response during 40-km cycling time-trial: relationship to muscle coordination and performance. J. Sci. Med. Sport 11, 363–370. 10.1016/j.jsams.2007.03.006 17703997

[B11] Bini R. R. Hume P. A. (2014). Biomechanics of cycling., in Springer Sci. eds Bini R. Carpes F (Springer International Publishing Switzerland). 10.1007/978-3-319-05539-8

[B12] Bini R. R. Hume P. A. Kilding A. E. (2014). Saddle height effects on pedal forces, joint mechanical work and kinematics of cyclists and triathletes. Eur. J. Sport Sci. 14, 44–52. 10.1080/17461391.2012.725105 24533494

[B13] Bini R. R. Hume P. A. Lanferdini F. Vaz M. (2013). Effects of moving forward or backward on the saddle on knee joint forces during cycling. Phys. Therapy Sport 14, 23–27. 10.1016/j.ptsp.2012.02.003 23312729

[B14] Blake O. M. Champoux Y. Wakeling J. M. (2012). Muscle coordination patterns for efficient cycling. Med. Sci. Sports Exer. 44, 926–938. 10.1249/MSS.0b013e3182404d4b 22089483

[B15] Briscoe M. J. Forgach M. S. Trifan E. Malek M. H. (2014). Validating the EMGFT from a single incremental cycling testing. Int. J. Sports Med. 35, 566–570. 10.1055/s-0033-1358672 24227121

[B16] Chapman A. R. Vicenzino B. Blanch P. Knox J. J. Dowlan S. Hodges P. W. (2008). The influence of body position on leg kinematics and muscle recruitment during cycling. J. Sci. Med. Sport 11, 519–526. 10.1016/j.jsams.2007.04.010 17719847

[B17] Cohen J. (1988). Statistical Power Analysis for the Behavioral Sciences. 2nd ed. (Hillsdale, NJ: L. Erlbaum Associates), xxi, 567.

[B18] Delp S. L. Hess W. E. Hungerford D. S. Jones L. C. (1999). Variation of rotation moment arms with hip flexion. J. Biomech. 32, 493–501. 10327003 10.1016/s0021-9290(99)00032-9

[B19] DeLuca C. Gilmore L. Kuznetsov M. Roy S. (2010). Filtering the surface EMG signal: movement artifact and baseline noise contamination. J. Biomech. 43, 1573–1579. 10.1016/j.jbiomech.2010.01.027 20206934

[B20] Dingwell J. Joubert J. Diefenthaeler F. Trinity J. (2008). Changes in muscle activity and kinematics of highly trained cyclists during fatigue. Trans. Bio-Med. Eng. 55, 2666–2674. 10.1109/TBME.2008.2001130 18990638 PMC2905840

[B21] Dorel S. Couturier A. Hug F. (2008). Intra-session repeatability of lower limb muscles activation pattern during pedaling. J. Electromyogr. Kinesiol. 18, 857–865. 10.1016/j.jelekin.2007.03.002 17449281

[B22] Dorel S. Couturier A. Hug F. (2009). Influence of different racing positions on mechanical and electromyographic patterns during pedaling. Scand. J. Med. Sci. Sports 19, 44–54. 10.1111/j.1600-0838.2007.00765.x 18266790

[B23] Dostal W. F. Soderberg G. L. Andrews J. G. (1986). Actions of hip muscles. Phys. Therapy 66, 351–361. 10.1093/ptj/66.3.351 3952148

[B24] Dunlap W. (1999). A program to compute McGraw and Wong's common language effect size indicator. Behav. Res. Methods Instr. Comp. 31, 706–709. 10.3758/BF03200750 10633989

[B25] Edwards R. Lippold O. (1956). The relation between force and integrated electrical activity in fatigued muscle. J. Physiol. 132, 677–681. 10.1113/jphysiol.1956.sp005558 13332603 PMC1363579

[B26] Erdemir A. McLean S. G. Herzog W. Van Den Bogert A. J. (2007). Model-based estimation of muscle forces exerted during movements. Clin. Biomech. 22:131–154. 10.1016/j.clinbiomech.2006.09.005 17070969

[B27] Ericson M. (1986). On the biomechanics of cycling. A study of joint and muscle load during exercise on the bicycle ergometer. Scand. J. Rehabil. Med. 16, 1–43. 3468609

[B28] Ettema G. Loras H. (2009). Effciency in cycling: a review. Euro. J. Appl. Physiol. 106, 1–14. 10.1007/s00421-009-1008-7 19229554

[B29] Faria E. W. Parker D. L. Faria I. E. (2005). The science of cycling: factors affecting performance. Part 2. Sports Med. 35, 313–37. 10.2165/00007256-200535040-00003 15831060

[B30] Farina D. Merletti R. Enoka R. M. (2004a). The extraction of neural strategies from the surface EMG. J. Appl. Physiol. 96, 1486–1495. 10.1152/japplphysiol.01070.2003 15016793

[B31] Farina D. Pozzo M. Merlo E. Bottin A. Merletti R. (2004b). Assessment of average muscle fiber conduction velocity from surface EMG signals during fatiguing dynamic contractions. IEEE Trans. Biomed. Eng. 51, 1383–1393. 10.1109/TBME.2004.827556 15311823

[B31a] Flaxman T. E. Alkjær T. Simonsen E. B. Krogsgaard M. R. Benoit D. L. (2017). Predicting the functional roles of knee joint muscles from internal joint moments. Med. Sci. Sports Exerc. 49, 527–537. 10.1249/MSS.0000000000001125 27755353

[B32] Fregly B. J. Zajac F. E. (1995). A state-space analysis of mechanical energy generation, absorption and transfer during pedaling. J. Biomech. 29, 81–90. 10.1016/0021-9290(95)00011-9 8839020

[B33] Gandevia S. C. (2001). Spinal and supraspinal factors in human muscle fatigue. Physiol. Rev. 81, 1725–1789. 10.1152/physrev.2001.81.4.1725 11581501

[B34] Gates D. H. Dingwell J. R. (2008). The effects of neuromuscular fatigue on task performance during repetitive goal-directed movements. Exp. Brain Res. 187, 573–585. 10.1007/s00221-008-1326-8 18327575 PMC2825378

[B35] Grappe F. (2009). Cyclisme et Optimisation de la Performance. Bruxelles: Deboeck Superieur.

[B36] Gregersen C. S. Hull M. L. (2003). Non-driving intersegmental knee moments in cycling computed using a model that includes three-dimensional kinematics of the shank/foot and the effect of simplifying assumptions. J. Biomech. 36, 803–813. 10.1016/S0021-9290(03)00014-9 12742448

[B37] Hautier C. A. Arsac L. M. Deghdegh K. Souquet J. Belli A. Lacour J. R. (2000). Influence of fatigue on EMG/force ratio and cocontraction in cycling. Med. Sci. Sports Exerc. 32, 839–843. 10.1097/00005768-200004000-00017 10776904

[B38] Hentschke H. Stüttgen M. C. (2011). Computation of measures of effect size for neuroscience data sets. Eur. J. Neurosci. 34, 1887–1894. 10.1111/j.1460-9568.2011.07902.x 22082031

[B39] Hermansen L. Saltin B. (1969). Oxygen uptake during maximal treadmill and bicycle exercise. J. Appl. Physiol. 26, 31–37. 10.1152/jappl.1969.26.1.31 5762873

[B40] Hermens H. J. Freriks B. Disselhorst-Klug C. Rau G. (2000). Development of recommendations for SEMG sensors and sensor placement procedures. J. Electromyogr. Kinesiol. 10, 361–374. 10.1016/S1050-6411(00)00027-4 11018445

[B41] Hirokawa S. (1991). Three-dimensional mathematical model analysis of the patellofemoral joint. J. Biomech. 24, 659–671. 10.1016/0021-9290(91)90331-G 1918090

[B42] Holliday W. Theo R. Fisher J. Swart J. (2019). Cycling: joint kinematics and muscle activity during differing intensities. Sports Biomech. 2, 1–15. 10.1080/14763141.2019.1640279 31475880

[B43] Hug F. Bendahan D. LeFur Y. Cozzone P. Grélot L. (2004a). Heterogeneity of muscle recruitment pattern during pedaling in professional road cyclists: a magnetic resonance imaging and electromyography study. Eur. J. Appl. Physiol. 92, 334–342. 10.1007/s00421-004-1096-3 15098128

[B44] Hug F. Decherchi P. Marqueste T. Jammes Y. (2004b). EMG versus oxygen uptake during cycling exercise in trained and untrained subjects. J. Electromyogr. Kinesiol. 14, 187–195. 10.1016/S1050-6411(03)00081-6 14962771

[B45] Hug F. Dorel S. (2009). Electromyographic analysis of pedaling: a review. J. Electromyogr. Kinesiol. 19, 182–198. 10.1016/j.jelekin.2007.10.010 18093842

[B45a] Hug F. Drouet J. M. Champoux Y. Couturier A. Dorel S. (2008). Interindividual variability of electromyographic patterns and pedal force profiles in trained cyclists. Eur. J. Appl. Physiol. 104, 667–678. 10.1007/s00421-008-0810-y 18629533

[B46] Hug F. Laplaud D. Lucia A. Grelot L. (2006). EMG threshold determination in eight lower limb muscles during cycling exercise: a pilot study. Int. J. Sport Med. 27, 456–462. 10.1055/s-2005-865787 16767610

[B47] Hug F. Laplaud D. Savin B. Grelot L. (2003). Occurrence of electromyographic and ventilatory thresholds in professional road cyclists. Eur. J. Appl. Physiol. 90, 643–646. 10.1007/s00421-003-0949-5 14508692

[B48] Jorge M. Hull M. L. (1986). Analysis of EMG measurements during bicycle pedaling. J. Biomech. 19, 683–694. 10.1016/0021-9290(86)90192-2 3793743

[B49] Karsten B. Jobson S. A. Hopker J. Stevens L. Beedie C. (2015). Validity and reliability of critical power field testing. Eur. J. Appl. Physiol. 115, 197–204. 10.1007/s00421-014-3001-z 25260244

[B50] Lenti M. De Vito G. Sbriccoli P. Palumbo A. S. Sacchetti M. (2010). Muscle fibre conduction velocity and cardiorespiratory response during incremental cycling exercise in young and older individuals with different training status. J. Electromyogr. Kinesiol. 20, 566–571. 10.1016/j.jelekin.2010.02.004 20202863

[B51] Lepers R. Hausswirth C. Maffiuletti N. Brisswalter J. van Hoecke J. (2000). Evidence of neuromuscular fatigue after prolonged cycling exercise. Med. Sci. Sports Exerc. 32, 1880–1886. 10.1097/00005768-200011000-00010 11079517

[B52] Li L. Caldwell G. E. (1999). Coefficient of cross correlation and the time domain correspondence. J. Electromyogr. Kinesiol. 9, 385–389. 10.1016/S1050-6411(99)00012-7 10597051

[B53] Lima da Silva J. Ekblom M. M. Tarassova O. Andersson E. Rönquist G. Grundström H.. (2018). Effect of increasing workload on knee extensor and flexor muscular activity during cycling as measured with intramuscular electromyography. PLoS ONE 13, 1–15. 10.1371/journal.pone.0201014 PMC6071990 30071032

[B54] Lucia A. Hoyos J. Pérez M. Santalla A. Chicharro J. L. (2002). Inverse relationship between VO2max and economy/efficiency in world-class cyclists. Med. Sci. Sports Exerc. 34, 2079–2084. 10.1097/00005768-200212000-00032 12471319

[B55] Lucia A. Pardo J. Durantez A. Hoyos J. Chicharro J. L. (1998). Physiological differences between professional and elite road cyclists. Int. J. Sports Med. 19, 342–348. 10.1055/s-2007-971928 9721058

[B56] Lucia A. Sánchez O. Carvajal A. Chicharro J. L. (1999). Analysis of the aerobic-anaerobic transition in elite cyclists during incremental exercise with the use of electromyography. Br. J. Sports Med. 3, 178–185. 10.1136/bjsm.33.3.178 10378070 PMC1756168

[B57] Lucia A. Vaquero A. F. Perez M. Sanchez O. Sanchez V. Gomez M. A.. (1997). Electromyographic response to exercise in cardiac transplant patients: a new method for anaerobic threshold determination? Chest 111, 1571–1576. 10.1378/chest.111.6.1571 9187176

[B58] Lukes R. A. Chin S. B. Haake S. (2005). The understanding and development of cycling aerodynamics. Sports Eng. 8, 59–74. 10.1007/BF02844004

[B59] Macdonald J. H. Farina D. Marcora S. M. (2008). Response of electromyographic variables during incremental and fatiguing cycling. Med. Sci. Sports Exer. 40, 335–344. 10.1249/mss.0b013e31815b491e 18202567

[B60] Martinez-Valdes E. Guzman-Venegas R. Silvestre R. MacDonald J. Falla D. Araneda O Haichelis D. (2016). Electromyographic adjustments during continuous and intermittent incremental fatiguing cycling. Scand. J. Med. Sci. Sports 26, 1273–1282. 10.1111/sms.12578 26493490

[B61] Midgley A. W. McNaughton L. R. Polman R. Marchant D. (2007). Criteria for determination of maximal oxygen uptake: a brief critique and recommendations for future research. Sports Med. 37, 1019–1028 10.2165/00007256-200737120-00002 18027991

[B62] Mokhtarzadeh H. Perraton L. Fok L. Muñoz A. Mario A. Clark R.. (2014). A comparison of optimization methods and knee joint degrees of freedom on muscle force predictions during single-leg hop landings. J. Biomech. 47, 2863–2268. 10.1016/j.jbiomech.2014.07.027 25129166

[B63] Neptune R. Kautz S. Hull M. (1997). The effect of pedaling rate on coordination in cycling. J. Biomech. 30, 1051–1058. 9391872 10.1016/s0021-9290(97)00071-7

[B64] Pataky T. C. Robinson M. A. Vanrenterghem J. (2013). Vector field statistical analysis of kinematic and force trajectories. J. Biomech. 46, 2394–2401. 10.1016/j.jbiomech.2013.07.031 23948374

[B65] Pinot J. Grappe F. (2014). Determination of maximal aerobic power on the field in cycling. J. Sci. Cycl. 3, 26–31.

[B66] Pouliquen C. Nicolas G. Bideau B. Garo G. Megret A. Delamarche P.. (2018). Spatiotemporal analysis of 3D kinematic asymmetry in professional cycling during an incremental test to exhaustion. J. Sports Sci. 30, 1–9. 10.1080/02640414.2018.1432066 29381424

[B67] Reiser R. F. Peterson M. L. Broker J. P. (2002). Influence of Hip orientation on wingate power output and cycling technique. J. Strength Condition. Res. 16, 566–60. 10.1519/1533-4287(2002)016<0556:IOHOOW>2.0.CO;2 12423185

[B68] Rouffet D. M. Hautier C. A. (2008). EMG normalization to study muscle activation in cycling. J. Electromyogr. Kinesiol. 18, 866–878. 10.1016/j.jelekin.2007.03.008 17507240

[B69] Ruby P. Hull M. Kirby K. Jenkins D. (1992). The effect of lower limb anatomy on knee loads during seated cycling. J. Biomech. 25, 1195–1207. 10.1016/0021-9290(92)90075-C 1400519

[B70] Sarre G. Lepers R. (2005). Neuromuscular function during prolonged pedaling exercise at different cadences. Acta Physiol. Scand. 185, 321–328. 10.1111/j.1365-201X.2005.01490.x 16266373

[B71] Sayers M. Tweddle A. Every J. Wiegand A. (2012). Changes in drive phase lower limb kinematics during a 60 min cycling time trial. J. Sci. Med. Sport 15, 169–174. 10.1016/j.jsams.2011.09.002 22018522

[B72] Sbriccoli P. V. Camomill A. Di Mario F. Quinzi F. Figura F. Felicini F. (2009a). Neuromuscular control adaptations in elite athletes: the case of top level karateka. Eur. J. Appl. Physiol. 108, 1269–1280. 10.1007/s00421-009-1338-5 20039054

[B73] Sbriccoli P. V. Sacchetti M. Felici F. Gizzi L. Lenti M. Scotto A.. (2009b). Non-invasive assessment of muscle fiber conduction velocity during an incremental maximal cycling test. J. Electromyogr. Kinesiol. 19, 380–386. 10.1016/j.jelekin.2009.03.008 19398350

[B74] Schmitz J. P. J. van Dijk J. P. Hilbers P. A. J. Nicolay K. Jeneson J. A. L. Stegeman D. F. (2012). Unchanged muscle fiber conduction velocity relates to mild acidosis during exhaustive bicycling. Eur. J. Appl. Physiol. 112, 1593–1602. 10.1007/s00421-011-2119-5 21861110 PMC3324688

[B75] Sinclair J. Hebron J. Atkins S. Hurst H. Taylor P. J. (2014). The influence of 3D kinematic and electromyographical parameters on cycling economy. Acta Bioeng. Biomech. 16, 91–97. 10.5277/ABB-00049-2014-02 25597361

[B76] Solomonow M. Barrata R. Zhou B. D'Ambrosia R. (1988). Electromyogram coactivation patterns of the elbow antagonist muscles during slow isokinetic movement. Exp. Neurol. 100, 470–477. 10.1016/0014-4886(88)90032-5 3366200

[B77] Suzuki S. Watanabe S. Homma S. (1982). EMG activity and kinematics cycling movements at different constant velocities. Brain Res. 240, 245–258. 10.1016/0006-8993(82)90220-7 7104687

[B78] Takaishi T. Yamamoto T. Ono T. Ito T. Moritani T. (1998). Neuromuscular, metabolic, and kinetic adaptations for skilled pedaling performance in cyclists. Med. Sci. Sports Exer. 30, 442–445. 10.1097/00005768-199803000-00016 9526892

[B79] Theurel J. Crepin M. Foissac M. Temprado J. E. (2011). Effects of different pedaling techniques on muscle fatigue and mechanical effciency during prolonged cycling. Scand. J. Med. Sci. Sports 22, 714–721. 10.1111/j.1600-0838.2011.01313.x 21507064

[B79a] Travis L. A. Arthmire S. J. Baig A. M. Goldberg A. Malek M. H. (2011). Intersession reliability of the electromyographic signal during incremental cycle ergometry: quadriceps femoris. Muscle Nerve. 44, 937–946. 22102465 10.1002/mus.22211

[B80] Turpin N. A. Guével A. Durand S. Hug F. (2011a). Effect of power output on muscle coordination during rowing. Eur. J. Appl. Physiol. 111, 3017–3029. 10.1007/s00421-011-1928-x 21451939

[B81] Turpin N. A. Guével A. Durand S. Hug F. (2011b). Fatigue-related adaptations in muscle coordination during a cyclic exercise in humans. J. Exp. Biol. 214, 3305–3314. 10.1242/jeb.057133 21900479

[B82] Umberger B. Martin P. (2001). Testing the planar assumption during ergometer cycling. J. Appl. Biomech. 17, 55–62. 10.1123/jab.17.1.55

[B83] van Bolhuis B. Gielen C. van Ingen Schenau G. (1998). Activation patterns of mono- and bi-articular arm muscle as a function of force and movement direction of the wrist in humans. J. Physiol. 508, 313–324. 10.1111/j.1469-7793.1998.313br.x 9490859 PMC2230856

[B84] Van Ingen Schenau G. J. Boots PJM de Groots G. Snackers R. J. van Woensel W. W. L. M. (1992). The constrained control of force and position in multi-join movements. Neuroscience 46, 197–207. 10.1016/0306-4522(92)90019-X 1594103

[B85] Wu G. Cavanagh P. (1995). ISB Recommendations for standardization in the reporting of kinematic data. J. Biomech. 28, 1257–1261. 10.1016/0021-9290(95)00017-C 8550644

[B86] Wu G. Siegler S. Allard P. Kirtley C. Leardini A. Rosenbaum D.. (2002). ISB recommendation on definitions of joint coordinate system of various joints for the reporting of human joint motion—part I: ankle, hip, and spine. J. Biomech. 35, 543–548. 10.1016/S0021-9290(01)00222-6 11934426

[B87] Zhang Y. Y. Johnson M. C. Chow N. Wasserman K. (1991). The role of fitness on VO_2_ and VCO_2_ kinetics in response to proportional step increases in work rate. Eur. J. Appl. Physiol. Occup. Physiol. 63, 94–100. 10.1007/BF00235176 1748111

